# P-2336. Efficacy of Treatment Regimens in Achieving Virological Response in Patients with Hepatitis D Virus Infection: A Network Meta-analysis

**DOI:** 10.1093/ofid/ofae631.2488

**Published:** 2025-01-29

**Authors:** Konstantinos Ouranos, Evangelia K Mylona, Charilaos Dellis, Dimitra Rafailia Bakaloudi, Fadi Shehadeh, Markos Kalligeros, Eleftherios Mylonakis

**Affiliations:** Houston Methodist Hospital, Houston, Texas; Houston Methodist Hospital, Houston, Texas; Houston Methodist Hospital, Houston, Texas; University of Washington, School of Medicine, Seattle, Washington; Houston Methodist Research Institute, Houston, TX, Houston, Texas; Warren Alpert Medical School of Brown University, Rhode Island Hospital, Providence, RI, Providence, Rhode Island; Houston Methodist Hospital, Houston, TX, Houston, Texas

## Abstract

**Background:**

Despite the availability of treatment options for hepatitis D virus (HDV) management, choosing the most appropriate drug scheme for achieving virological response (VR) has not been adequately studied.

**Figure:**
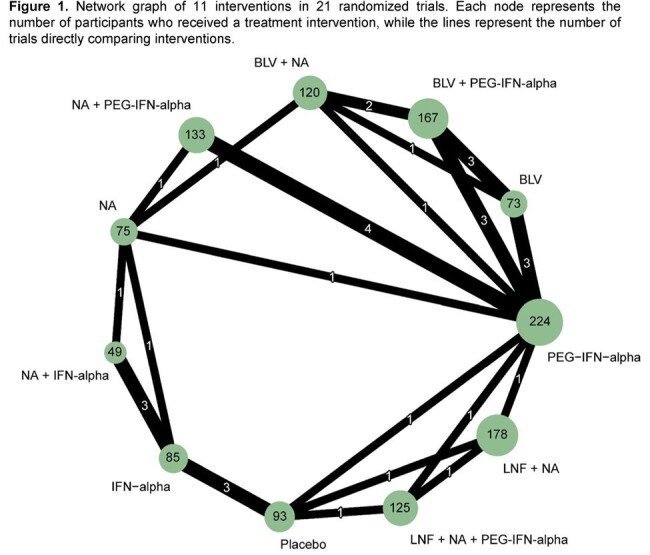

**Methods:**

We conducted a systematic review and network meta-analysis of randomized trials in PubMed, EMBASE, and Web of Science databases reporting rates of VR (defined as undetectable HDV RNA or decrease by ≥ 2 log_10_ IU/ml at the end of treatment compared with baseline measurements). Available treatment regimens included interferon-alpha (IFN-alpha), pegylated IFN-alpha (PEG-IFN-alpha), nucleoside analogs (NA), bulevirtide (BLV), lonafarnib (LNF), combination therapy or placebo. We used random effects model and estimated odds ratio (OR) with 95% confidence interval (CI) to compare treatments based on VR. To identify the best treatment, we estimated the surface under the cumulative ranking (SUCRA) curve. Analyses were performed using the *meta* and *netmeta* packages in R (version 4.3.3).

**Figure:**
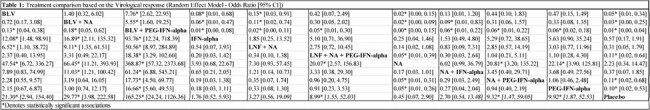

*Denotes statistically significant associations

**Results:**

A total of 1322 patients from 21 studies were included in the analysis and were randomized in one of 11 treatment interventions, as described in Figure 1. Treatment duration ranged from 24 to 96 weeks. BLV + PEG-IFN-alpha was ∼6 times more likely to result in VR compared with BLV alone (OR, 5.55 [95%CI: 1.60, 19.25]). Treatment with BLV + PEG-IFN-alpha was ∼17 times more likely to result in VR compared with PEG-IFN-alpha monotherapy (OR, 16.7 [95%CI: 5.6, 49.5]) (Table 1), the main agent recommended by official guidelines for HDV management. Based on probability ranking, the most effective drug schemes for achieving VR at the end of treatment were BLV + PEG-IFN-alpha (99.7%), BLV + NA (84.4%), BLV (78%), and PEG-IFN-alpha (61.3%).

**Conclusion:**

BLV + PEG-IFN-alpha is superior in achieving VR at the end of HDV treatment compared with all other available drug schemes. Further research to determine whether BLV + PEG-IFN-alpha can maintain long-term VR after end of treatment is needed. Studies should also evaluate the impact of different BLV dosages on VR.

**Disclosures:**

Eleftherios Mylonakis, MD, PhD, BARDA: Grant/Research Support|Basilea: Advisor/Consultant|Chemic Labs/KODA Therapeutics: Grant/Research Support|Cidara: Grant/Research Support|Leidos Biomedical Research Inc./NCI: Grant/Research Support|NIH SciClone Pharmaceuticals: Grant/Research Support|NIH/NIAID: Grant/Research Support|NIH/NIGMS: Grant/Research Support|Pfizer: Grant/Research Support|Regeneron Pharmaceuticals, Inc.: Grant/Research Support

